# Clinical characteristics and prognostic analysis of multiple primary malignant neoplasms in female patients with breast cancer or genitalia malignancies

**DOI:** 10.7717/peerj.13528

**Published:** 2022-06-24

**Authors:** Li Xiao, Tiantian Cao, Jiali Ou, Weijiang Liang

**Affiliations:** 1 Medical Oncology, Nanfang Hospital, Southern Medical University, Guangzhou, Guangdong, China; 2 Intensive Care Unit, Nanfang Hospital, Southern Medical University, Guangzhou, Guangdong, China

**Keywords:** Multiple primary malignant neoplasms, Female, Breast cancer, Genitalia malignancies

## Abstract

**Background:**

As public awareness of health has increased and diagnostic and treatment options have improved, the survival of patients with malignant tumors has continued to extend, and the population has been aging, the number of multiple primary malignant neoplasms has gradually increased in recent years. There are few reports concerning female patients with multiple primary malignant neoplasms of breast cancer or genitalia malignancies. In this study, we aimed to analyze the clinical characteristics and prognosis of multiple primary malignant neoplasms in female patients with breast cancer or genitalia malignancies, as well as further explore the factors that affect the survival.

**Methods:**

We collected clinical data on 80 female patients diagnosed with multiple primary malignant neoplasms of the breast or genitalia, described their clinical features. Furthermore, we calculated the survival and prognostic factors for 52 participants.

**Results:**

In our study, the prevalence rate of multiple primary malignant neoplasms was 0.66% (367/55404). Corresponding to female patients with multiple primary malignant neoplasms of breast cancer or genitalia malignancies, it was 1.4% (80/5707). the median age of diagnosis for the first tumor was 48 years, and the median age of diagnosis for the second tumor was 52 years. Regarding the interval, 67.57% (50/74) of patients were within five years. Most tumors were located in the breast (44.68%), followed by the uterus (20.21%), the ovary (17.02%), and the cervix (15.96%). The overall 12-, 36-and 60-month survival rates of the patients were 86.4%, 74.3%, and 69.8%. For the female patients, the stage (III–IV) (*P* = 0.046), non-radical surgery (*P* = 0.002), and types of the last tumor (breast cancer or genitalia malignancies) (*P* = 0.019) were associated with the poor prognosis.

**Conclusions:**

Female patients with breast cancer or genital malignancies should pay attention to screening for the second tumor, especially within 4 years after the first tumor diagnosed. Furthermore, during tumor screening, it may be recommended for these patients to focus on colorectal cancer and lung cancer. Compared with previous studies, in addition to clinical staging and types of surgery, we found whether the last tumor was breast cancer or genitalia malignancies should also be considered a prognostic factor.

## Introduction

Multiple primary malignant neoplasms (MPMNs) are defined as two or more malignant tumors of distinct histological types in the same individual simultaneously or successively. MPMNs may be classified into two subtypes: synchronous multiple primary malignant neoplasms (SMPMNs) and metachronous multiple primary malignant neoplasms (MMPMNs). When the second primary malignancy is diagnosed within six months of the first primary malignancy diagnosis, it may be considered SMPMNs. Otherwise, it may be MMPMNs (Moertel, Dockerty & Baggenstoss, 1961). As public awareness of health has increased and diagnostic and treatment options have improved, the survival of patients with malignant tumors has continued to extend, and the population has been aging, the number of MPMNs has gradually increased in recent years (Mariotto et al., 2007). The etiology of MPMNs remains unknown and may be related to factors such as environment, lifestyle (smoking, drinking, obesity), gene, immunity, endocrine and treatment (chemotherapy, radiotherapy), *etc* (Haas et al., 1987; Harvey & Brinton, 1985; SprattJr & Hoag, 1966; Varol et al., 2018; Yamamoto et al., 2006). At present, reports of MPMNs mainly focus on head and neck tumors, digestive tumors, and lung cancer. There are few reports concerning female patients with multiple primary malignant neoplasms of breast cancer or genitalia malignancies. Due to a lack of experience in diagnosing and treating these patients in clinical work, some may be misdiagnosed as recurrence and metastasis of primary malignant tumors. Therefore, it may be of significance to investigate the clinical characteristics and prognosis of these patients.

In this study, we aimed to analyze the clinical characteristics and prognosis of multiple primary malignant neoplasms in female patients with breast cancer or genitalia malignancies and further explore the factors that affect the survival.

## Materials & Methods

In 1932, Warren and Gates (Warren, 1932) proposed the diagnostic criteria for MPMNs: (1) Each tumor is confirmed to be malignant by pathology; (2) Each tumor has its unique pathological morphology; (3) Each tumor must be excluded as recurrence or metastasis; (4) Each tumor must occur in a different and discontinuous of the site. In 2005, The International Rules for MPMNs supplemented this standard: different morphologic neoplasms should be regarded as multiple primary malignant neoplasms, even if they are diagnosed simultaneously at the same site (Working Group Report, 2005). Firstly, through the data statistics department and the doctor’s work system, we screened out patients with multiple primary malignant neoplasms who met the above diagnostic criteria and were admitted to Nanfang Hospital, Southern Medical University, from January 2008 to February 2018. Secondly, among the selected patients, we screened out female patients with one or more tumors being breast cancer or genitalia malignancies (such as uterus, ovary, vagina, *etc*.). Finally, we collected the patient’s tumor-related information and other information such as family history, congenital diseases, history of underlying diseases, menopause, and date of last outpatient visit. The tumor diagnosed firstly was defined as the first tumor, the second tumor was the one diagnosed secondly, and the last tumor was the tumor diagnosed finally. First of all, we collected clinical data on 80 female patients and described their clinical features. Then, we performed a prognostic analysis on 52 patients whose last tumor has a precise diagnosis time, stage, and treatment according to the exclusion criteria. The exclusion criteria for the prognostic analysis: (1) The last tumor was a hematological tumor with non-solid tumor and an unknown stage; (2) the last tumor was a malignant tumor with an unspecified stage undergoing surgery in other hospitals; (3) patient with other causes of death: such as cardiovascular disease, *etc*. The AJCC 7th edition malignancies clinical stages were used for staging. Fifty-two patients were followed up through telephone and medical records until March 30, 2021, or death. Overall survival (OS) was computed from diagnosis of the last tumor to death or the last follow-up. This study was given the stamp of approval by the medical ethics committee of Nanfang Hospital, Southern Medical University (NO.: NFEC-2021-219), and individual consent for this analysis was waived.

The SPSS (version 25.0; IBM, Armonk, NY, USA) program and R software (R Core Team, 2020) were used for statistical analysis. The median value and the range were used to represent non-normally distributed data. Fisher exact test and chi-square test were applied to test for tumor associations. The Kaplan–Meier curves were applied to compare the OS of different groups, and the log-rank test was utilized to verify statistical significances. Independent prognostic factors were explored by the performance of univariate and multivariate Cox regression analysis. *P* values < 0.05 were considered to be significant.

## Results

From January 2008 to February 2018, the Nanfang Hospital, Southern Medical University admitted 55,404 patients with primary malignancies, 367 of whom had MPMNs, for the prevalence rate of 0.66% (367/55404). During the same period, 5707 patients with breast cancer or genitalia malignancies were admitted, 80 of whom were diagnosed with MPMNs (including 75 double primary malignancies, five triple primary malignancies), the prevalence rate of 1.4%.

**Figure 1:**
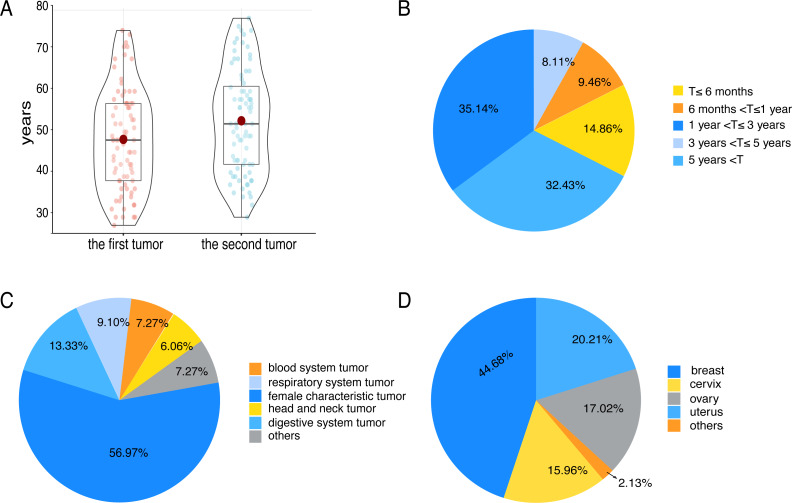
Some clinical characteristics of multiple primary malignant neoplasms in female patients with breast cancer or genitalia malignancies. (A) The tumor diagnosis age of 80 patients. (B) The diagnosis interval (T) distribution of 74 patients. (C) The tumor sites distribution. Others including renal carcinoma, skin cancer, pelvic sarcoma and thymic carcinoma. (D) The locations distribution of breast cancer and genitalia malignancies. Others including vaginal carcinoma and vulvar cancer.

In 80 patients, the median age of diagnosis for the first tumor was 48 years (range 27–75), and the median age of diagnosis for the second tumor was 52 years (range 29–78) (Fig. 1A). The median age of patients with their first tumor being breast cancer or genitalia malignancies was 47 years. Correspondingly, the median age of patients whose first tumor was non-breast cancer or non-genitalia malignancies was 52 years. There were 74 cases with a precise diagnosis interval (T), 14.86% of which suffered from SMPMNs, and 85.14% suffered from MMPMNs. The data showed that 67.57% of patients whose diagnosis interval was within 5 years (Fig. 1B).

We found that nine patients had a positive family history of cancer, including 8 first-degree relatives and one second-degree relative. Eighteen patients had a history of hypertension, diabetes, coronary heart disease, hyperthyroidism, or cerebral infarction. When the first tumor was diagnosed, 37 patients were in premenopause, 31 patients were in natural menopause, eight patients were in unnatural menopause. Similarly, when the second tumor was diagnosed, 20 patients were in premenopause, 34 patients were in natural menopause, and 22 patients were in unnatural menopause.

We observed 63 participants whose first tumors were breast cancer or genitalia malignancies. Relatively, 30 patients were found to have second tumors that were breast cancer or genitalia malignancies. Eleven patients had two primary malignancies, which were all breast cancer or genitalia malignancies, and three patients had three primary malignancies, two of which were breast cancer or genitalia malignancies. In terms of the location of tumors, the most common were breast cancer and genitalia malignancies (56.97%) (Fig. 1C), followed by the digestive system tumors, and the respiratory system tumors. As for breast and genitalia malignancies, the majority were breast cancer (44.68%) (Fig. 1D). The most common associations were cervical-lung cancer (*n* = 7) (*P* = 0.006), followed by breast-colorectal cancer (*n* = 6) (*P* = 0.666), breast-lung cancer (*n* = 5) (*P* = 0.394), endometrial-colorectal cancer (*n* = 5) (*P* = 0.291), and ovary-colorectal cancer (*n* = 4) (*P* = 0.490).

Most of our patients got a pathological diagnosis through surgery. From the detailed pathological data of 121 solid tumors, the most frequent pathology types were adenocarcinomas (81/121) and squamous carcinoma (24/121). The remaining were sarcoma, clear cell carcinoma, *etc*. In adenocarcinoma, breast cancer (24/81) was the most common type, followed by endometrial cancer (14/81) and colorectal cancer (14/81). The rest were ovarian cancer (11/81), lung adenocarcinoma (11/81), thyroid cancer (4/81), stomach cancer (2/81), and cholangiocarcinoma (1/81). Cervical cancer (9/24) was the most common type of squamous carcinoma, followed by lung squamous cell carcinoma (5/24), nasopharyngeal carcinoma (3/24), esophageal cancer (3/24), and others (vaginal cancer, thymic cancer, skin cancer) (4/24).

While all of our patients accepted treatment after the diagnosis of their first tumor, 9 did not receive any form of anti-tumor therapy after the diagnosis of their second tumor, including surgery, chemotherapy, or radiotherapy. For the first tumors, 88.75% (71/80) of patients underwent surgery, 31.25% (25/80) received surgery alone, 52.50% (42/80) were treated with surgery combined with chemotherapy, and only 11.25% (9/80) did not accept operation. In chemotherapy, 58.75% (47/80) of patients received chemotherapy, and 17.50% (14/80) were treated with chemotherapy combined with radiotherapy. In addition, 27.50% (22/80) received radiotherapy. For the second tumors,72.50% (58/80) of patients underwent surgery, 23.75% (19/80) received surgery alone, and 41.25% (33/80) were treated with surgery combined with chemotherapy. About chemotherapy, 63.75% (51/80) of patients received chemotherapy, and 13.75% (11/80) were treated with chemotherapy combined with radiotherapy. Moreover, only 13.75% (11/80) of patients received radiotherapy for primary tumors or metastatic tumors.

**Figure 2:**
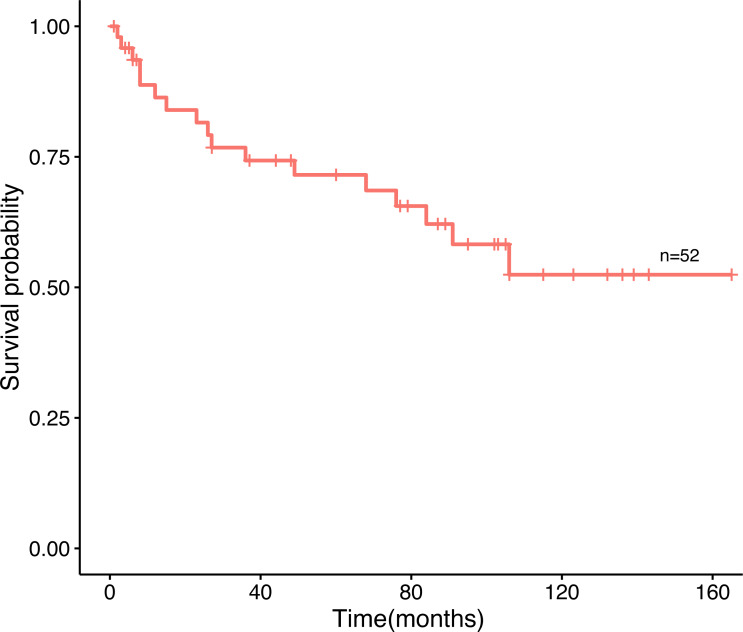
The overall survival curve. The survival curves of 52 patients.

**Figure 3:**
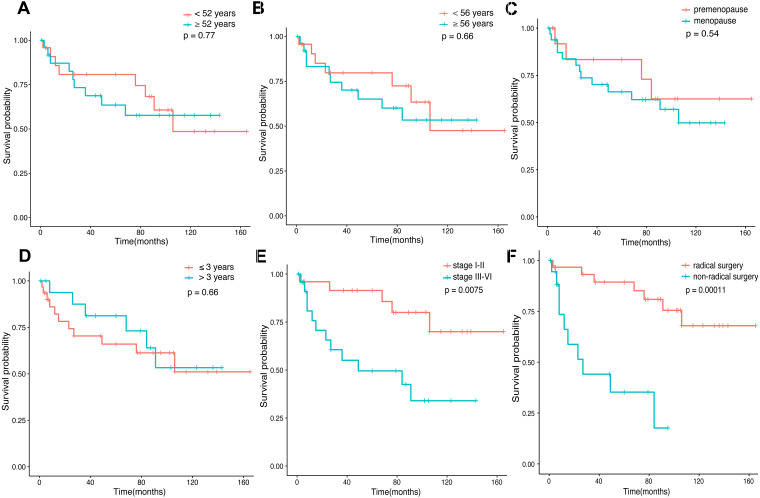
Survival time in connection with several groups. (A) The survival time of age at the first tumor diagnosis for the patients. (B) The survival time of age at the last tumor diagnosis for the patients. (C) The survival time of the menstrual status (premenopause *vs.* menopause) at the first tumor diagnosis for the patients. (D) The survival time of interval ≤3 years or >3 years for the patients. (E) The survival time of the last tumor stage (I–II *vs.* III–IV) for the patients. (F) The survival time of surgery types (radical *vs.* non-radical) of the last tumor for the patients.

Amid the 80 patients, 52 patients selected for prognostic analysis were followed up until March 30, 2021, with a median follow-up of 54.5 months (range 1–165 months). By the time the follow-up was complete, 17 patients had died, 22 had survived, and 13 had been lost to follow-up. The median age of diagnosis for the first tumor was 52 years old, and 56 years old for the last tumor. The most common tumor associations were cervical-lung cancer (*n* = 7), followed by breast-colorectal cancer (*n* = 5), endometrial-colorectal cancer (*n* = 4), breast-endometrial cancer (*n* = 3), and breast-nasopharyngeal cancer (*n* = 3). The overall 12-, 36-and 60-month survival rates of the 52 patients were 86.4%, 74.3%, and 69.8% (Fig. 2). In our analysis, whether the median age of the first tumor was ≥ 52 or not, and whether the median age of the last tumor was ≥ 56 or not, there was no statistical distinction in survival time (*P* = 0.77, *P* = 0.66, Fig. 3A, Fig. 3B). Similarly, there was no statistical discrepancy in survival based on the menstrual status at the time of the first tumor diagnosis. (*P* = 0.54, Fig. 3C). Moreover, we analyzed whether there was a statistical difference in survival time of interval ≤ 3 years or not, and the result indicated no difference (*P* = 0.66, Fig. 3D). Patients whose last tumor stage was I–II had a longer survival time than those whose last tumor stage was III–IV (*P* = 0.0075, Fig. 3E), and Patients whose last tumor accepted radical surgery had a longer survival time than those whose last tumor accepted non-radical surgery (*P* = 0.00011, Fig. 3F).

In univariate logistic regression analysis, stage (III–IV) (*P* = 0.012) and non-radical surgery (*P* = 0.00057) were found to be significantly associated with survival. As well, we found the stage (III–IV) (*P* = 0.046), non-radical surgery (*P* = 0.002), and types of the last tumor (breast cancer or genitalia malignancies) (*P* = 0.019) were all poor prognostic factors connected with survival based on the multivariate analysis (Table 1).

**Table 1:** Univariate and multivariate analysis for the survival of 52 patients.

	Univariate			Multivariate		
	HR	95% CI	*P*-value	HR	95% CI	*P*-value
The first tumor diagnosis age						
<52 years						
≥52 years	1.2	(0.44–3)	0.77			
Menstrual status at the first tumor diagnosis						
premenopause						
menopause	1.4	(0.46–4.4)	0.54			
The last tumor diagnosis age						
<56 years						
≥56 years	1.2	(0.47–3.3)	0.66			
The last tumor stage						
I–II						
III–IV	2	(1.2–3.3)	0.012	1.84	(1.011–3.36)	0.046
The last tumor surgery types						
radical						
non-radical	6.4	(2.2–18)	0.00057	7.56	(2.163–26.43)	0.002
The last tumor types						
breast or genitalia						
non-breast or non-genital	0.82	(0.32–2.1)	0.68	0.27	(0.089–0.81)	0.019
Interval time						
≤3 years						
>3 years	0.8	(0.3–2.2)	0.66			

## Discussion

In recent years, patients with malignancies have been prolonged, and the risk of developing MPMNs has gradually increased. Research has shown there was a difference in the prevalence rate of MPMNs. Compared with 0.73%–11.7% in other countries (Demandante, Troyer & Miles, 2003), the prevalence of MPMNs in China was about 0.47%–2.5% (Li, Zhan & Li, 1996; Wang et al., 2019) The incidence we counted was within the range of the ratio reported in China, but lower than the rate reported by Motuzyuk (Motuzyuk et al., 2018). Our study was a single-center study, and the majority of the enrolled patients were from Guangdong Province, and the majority of patients enrolled were from Guangdong Province, where they were exposed to a different environment in terms of diet and climate than patients in other reports. Additionally, the difference was due to limited diagnostic methods and experience with MPMNs. We found several suspicious patients who did not undergo pathological biopsies due to their physical or family economic conditions throughout the study. Only some relevant imaging confirmed the existence of the tumors that did not meet the current MPMNs diagnostic criteria.

Carcinogenesis is a multi-step, long-term procedure, and as life expectancy enhances, the possibility of being diagnosed with cancer increases as well. As a result, the risk of developing MPMNs gradually increases with age (Luciani et al., 2009). Additionally, patients with cancer had a much higher risk of developing the second primary malignancy than the general population. About half of the patients were menopausal when the first tumor was diagnosed, and more than 70% of the patients were menopausal when the second tumor was diagnosed. However, little research exists to show whether menopausal women are more likely to develop MPMNs. Our study found significantly more patients with MMPMNs than SMPMNs, which was consistent with reported before (Aydiner et al., 2000). The median age of diagnosis for the first tumor was 48 years, and the median age of diagnosis for the second tumor was 52 years. Moreover, 67.57% of patients developed the second tumors within 5 years after the first tumors were diagnosed. It is reasonable to believe that the second tumor may be found within 4 years of the diagnosis of the first tumor, which was different from the literature reported (Utada et al., 2014). Consequently, female patients with breast cancer or genital malignancies should pay attention to screening for the second tumor, especially within 4 years after the first tumor diagnosed, to avoid misdiagnosis or missed diagnosis and achieve early detection, early diagnosis, and early treatment to prolong survival. We observed that a small percentage of patients had a family history of cancer. This may be explained by the reported hereditary tumor syndrome. Hereditary breast and ovarian cancer syndrome (HBOC) is a hereditary tumor that can be definitively diagnosed by the detection of a germline mutation of the BRCA1 or BRCA2 gene (Noh et al., 2008). Women who carry mutations in the BRCA1 and BRCA2 genes have a substantially increased risk of developing breast cancer and ovarian cancers as compared with the general population (Neuhausen et al., 2009).

Owing to the factors of ethnicity, regions and environmental exposure, it is diverse that the current reports on the prone sites of MPMNs for all people regardless of gender. For females worldwide, breast cancer is currently the most common malignant tumor. Similarly, breast cancer has become the most common malignant tumor in Chinese women, accounting for 12.2 percent of newly diagnosed cases and 9.6 percent of all breast cancer deaths worldwide (Fan et al., 2014). In our study, the breast was the most frequent site, which was consistent with the research above. Additionally, the digestive system, respiratory system, and blood system were found generally. This corresponded with the prone sites mentioned in certain studies (Babacan et al., 2012; Lv et al., 2017; Zhai et al., 2018). We found hematological tumors mostly appeared within 1-3 years after the diagnosis of the first tumors which received chemotherapy. Research demonstrated that the relative risk of acute leukemia after treatment with cyclophosphamide alone was obviously increased (*P* < 0.05), at 14.6 for ovarian cancer patients and 2.7 for breast cancer patients (Haas et al., 1987). Therefore, in the choice of chemotherapy for the tumor, we should try to choose safer and more effective drugs to reduce the incidence of leukemia after chemotherapy. In our data, 14 patients had two or more breast cancers or genitalia malignancies, confirming that the associations between the breast, uterus, and ovary were extremely strong (Tabuchi et al., 2012). In our study, among non-breast or non-genital malignancies, the most common was the digestive system tumors, followed by the respiratory system tumors. There were 15 patients with colorectal cancer and 15 patients with lung cancer. Our study indicated a correlation between cervical cancer and lung cancer (*P* = 0.006). Previous studies have shown an increased risk of cervical cancer and lung cancer with mutations in the LKB1 gene (Sanchez-Cespedes, 2007; Wingo et al., 2009). In addition, some studies have confirmed that breast cancer or genitalia malignancies were closely related to lung cancer and colorectal cancer (Izmajłowicz, Kornafel & Błaszczyk, 2014; Luciani et al., 2009; Zhai et al., 2018). A prospective cohort study based on a female breast cancer population showed that the SIR of secondary primary colorectal cancer among breast cancer survivors was 1.59 (Lu et al., 2016) BRCA mutations heighten the risk of developing breast cancer. Moreover, it has been confirmed that BRCA1 mutations also raise the risk of colorectal cancer (Phelan et al., 2014). Similarly, mutations in mismatch repair genes such as MSH2, MSH6, MLH1, PMS1, and PMS2 may increase the risk of endometrial, ovarian, and colorectal cancers, resulting in Lynch syndrome (Bonadona et al., 2011). However, there was no evidence in our study to confirm a strong association between breast, endometrial and ovarian cancers and colorectal cancer. The small sample size of our study could account for this outcome.

Currently, existing treatments for MPMNs predominantly depend on the stage, pathological type, and tolerability of patients. Priority should be given to treating tumors that are adverse to the patient’s survival or quality of life. The prognosis of patients receiving operations based on comprehensive treatment is superior to that of operation alone (Lv et al., 2017). The prognosis was miserable when the best supportive treatment was given only without anti-tumor therapy (Kim et al., 2015). Our results demonstrated that surgery for the first tumor was routine. The second tumor was treated primarily with surgery and chemotherapy. It is worth noting that radiotherapy only accounted for a small proportion, which could be attributed to the imperfect system, the shortage of radiotherapy physicians, and defective equipment at that time (Yan et al., 2021). As previously reported by some researchers, all factors affecting the prognosis of patients with MPMNs, including age, the number of tumors, the interval between tumors, the distribution of lesions, stage, and treatment, are related (Ha et al., 2007; Jiang et al., 2019; Kim et al., 2018; Kim et al., 2015; Lee et al., 2015; Lv et al., 2017; Motuzyuk et al., 2018). The longer the interval, the better the prognosis (Kim et al., 2018). The second primary cancer had a more significant impact on the prognosis than gastric cancer itself for patients of gastric cancer with the second primary cancer (Ha et al., 2007). The second or the last tumor is more critical for survival. Based on this, we conducted a multivariate analysis. Compared with previous studies, besides clinical staging and types of surgery, whether the last tumor was breast cancer or genitalia malignancies should also be considered a prognostic factor. This may be due to the fact that some genitalia malignancies, such as ovarian cancer, lack obvious early clinical manifestations, making early detection difficult, leaving some patients at risk of missing the optimal time for treatment and thus having a poor prognosis. Zhao et al. (2015) reported that a female had a good quality of life after being diagnosed with eight primary malignant neoplasms in China. Patients with MPMNs may have a desirable prognosis if sufficient diagnosis, treatment, and long-term regular follow-up are rigorously performed.

Nonetheless, our study has several limitations. Firstly, patients were recruited exclusively from Nanfang Hospital, Southern Medical University, and small cases. Secondly, no research on the pathogenic factors was conducted. Finally, the impact of the first tumor staging and types of surgery on the prognosis was not tracked. We expect that more research could be carried out deeply in the future.

## Conclusions

Female patients with breast cancer or genital malignancies should pay attention to screening for the second tumor, especially within 4 years after the first tumor diagnosed. Furthermore, during tumor screening, it may be recommended for these patients to focus on colorectal cancer and lung cancer. Compared with previous studies, in addition to clinical staging and types of surgery, we found whether the last tumor was breast cancer or genitalia malignancies should also be considered a prognostic factor. Additionally, sufficient diagnosis, treatment, and long-term regular follow-up have been highly significant for patients with malignancies.

## Supplemental Information

10.7717/peerj.13528/supp-1 Supplemental Information 1 Raw data

10.7717/peerj.13528/supp-2 Supplemental Information 2 The number of tumors in survival

## Additional Information and Declarations

### Data Availability

The following information was supplied regarding data availability: The raw measurements are available in the Supplementary Files.
