# Supplementary figures and images for: Clinical characteristics and prognostic analysis of multiple primary malignant neoplasms in female patients with breast cancer or genitalia malignancies
Source: PeerJ. 2022 Jun 24;10:e13528. doi: 10.7717/peerj.13528 (PMC9235813; doi:10.7717/peerj.13528)

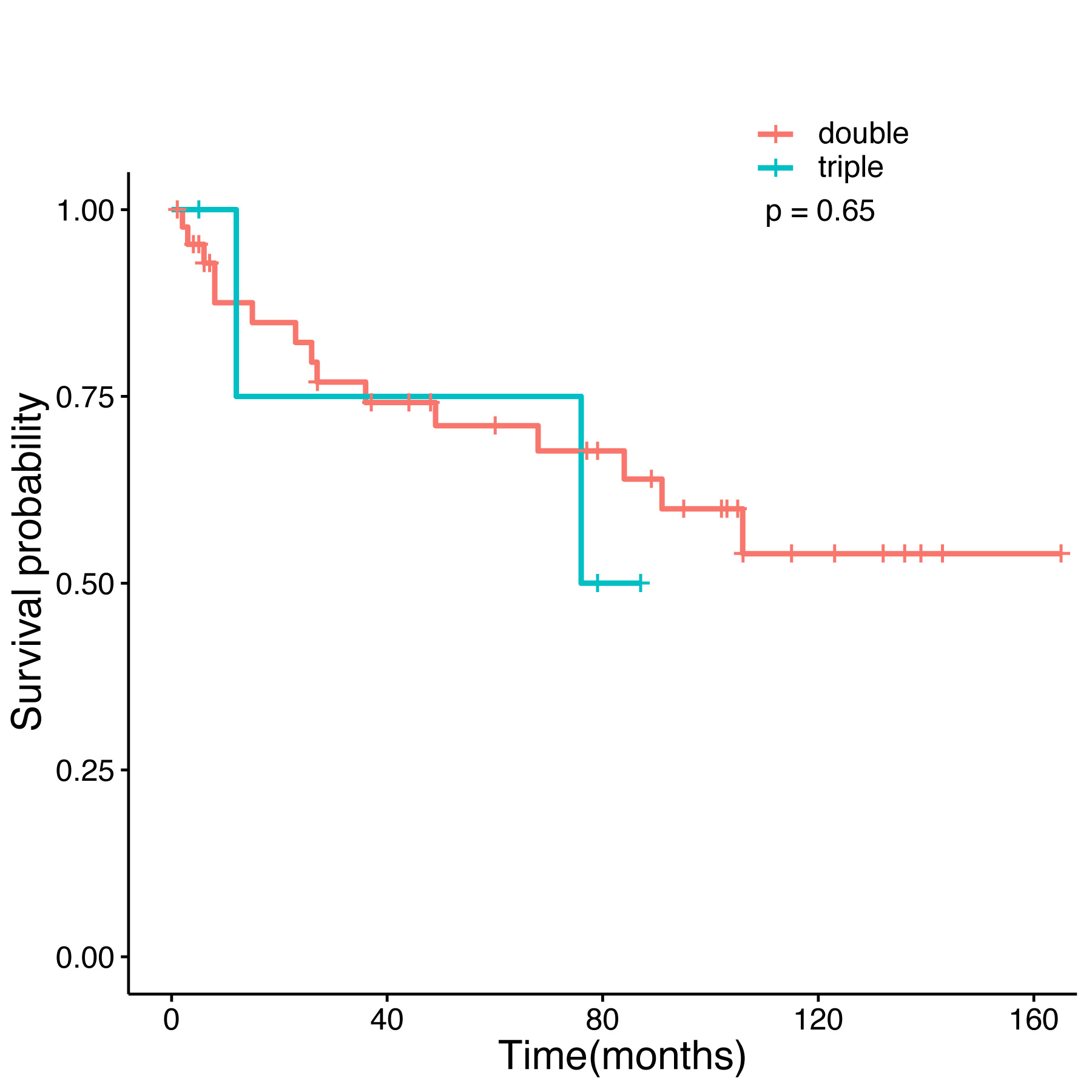

Supplement: Supplemental Information 2 [file peerj-10-13528-s002.jpg]
